# A metabolic test to distinguish temporary and persistent renal graft dysfunction prior to transplantation

**DOI:** 10.1038/s43856-026-01736-x

**Published:** 2026-07-09

**Authors:** Charlotte von Horn, Laura Malkus, Ruth Winzen, Thomas Minor

**Affiliations:** Surgical Research Department, University Hospital Essen, Essen, Germany

**Keywords:** Kidney, Predictive markers

## Abstract

**Background:**

Despite donor organ shortage, many grafts are still discarded due to questionable quality. Functional evaluation of the graft prior to transplantation is compromised by the fact that many kidneys suffer from transient filtration failure early after preservation. We tried to circumvent this problem by using a non-invasive quantitative method to determine metabolic turnover of 13C-acetate in the isolated perfused kidney and then correlating this to graft function after actual transplantation.

**Methods:**

Female porcine kidneys subjected to varying degrees of ischemic injury were put on a machine perfusion device. After injection of non-radioactive 13C-labelled acetate to the perfusate, its turnover by the citrate cycle was quantified by direct detection of 13CO2 in the gas outflow of the oxygenator with a mobile, high precision laser spectrometer.

**Results:**

Even in the absence of urine production the method discriminates all kidneys that go on to resume function from those that do not resume function after actual transplantation. There is no overlap between the two groups.

**Conclusions:**

Potentially the 13 C turnover test offers an objective, quantitative and valuable adjunct in the toolbox for pre-transplant evaluation of questionable grafts.

## Plain language summary

Despite the shortage of donor organs, many grafts are discarded as it is unclear whether they will function properly following transplant. A measure of whether an organ is functioning is metabolic turnover of acetate. We measured this in kidneys removed from pigs using a radioactively labelled tracer. We were able to distinguish between kidneys that resumed function after the actual transplantation and those that did not. Thus, this test appears to have the potential to be used to determine which donor organs are most likely to work well following transplantation before they are transplanted. Clinically this could reduce organ failure and also enable more donor organs to be used, as some that would previously have been discarded can be demonstrated to still be functioning.

## Abstract (web summary)

von Horn et al. describe a method to assess organ quality prior to transplantation based on a kidney’s metabolic function. The test, which is performed during isolated organ perfusion, distinguishes between pig grafts that resume function or fail upon subsequent transplantation in vivo.

## Introduction

Functional evaluation of donor graft quality prior to transplantation has become an increasingly important challenge in transplantation medicine. Due to the persisting shortage of donor organs, the criteria for organ donation had to be extended such that grafts could be retrieved even from older donors, including frequent co-morbidities as well as from donors who’s heart had stopped beating prior to explantation^1,2^. However, although such grafts did function satisfactorily in the donor, they are often afflicted by a reduced tolerance to storage and transport outside the organism, which may adversely affect parenchymal function and result in limited or even absent organ function after transplantation^3^. With regard to the kidney, the situation is further complicated by the fact that renal diuresis is often compromised early after ischemic preservation, although later recovery of renal filtration and glomerular function can be observed in most of the cases later after transplantation^4,5^. It hence follows that functional evaluation of renal filtration prior to transplantation, e.g., by extracorporeal machine perfusion, may easily identify the fraction of immediately well-functioning organ grafts^6,7^, but falls short of telling those organs that only suffer from transient deficits in diuresis and would resume function within several days. If these grafts, that may thus be as well considered to be transplanted, could be separated from the permanently non-functioning kidneys, the voluntary discard of only temporarily anuric kidneys could be prevented, and the shortage of donor organs alleviated. One approach to assess renal integrity in the absence of diuresis has been sought in the detection of biological markers that are released into the perfusate upon machine perfusion of the kidney. Although some of these biomarkers did show a certain correlation with ulterior renal function after transplantation, the decision for acceptance or discard of a single graft in question may not be based on any of these parameters^8,9^. In search for a more decisive parameter of renal viability, we have developed a method to evaluate the metabolic activity of the grafts by noninvasive measurement of the turnover rate in the citric acid cycle upon isolated machine perfusion. Machine perfusion of individual donor kidneys is often performed at lower temperatures^10^, which significantly simplifies the technical requirements as well as the demands on the perfusion medium^11,12^, while perfusion at normothermia is a rather complex procedure, and the putative need of erythrocytes as oxygen carriers has yet widely hindered its translation into clinical practice^11,13^. Unfortunately, viability assessment below normothermic temperatures is not currently well defined^9,14,15^. But then, it has recently been shown by De Haan and coworkers that the metabolic activity of the citric acid cycle is steadily operative even at sub normothermia^11^. We previously confirmed the concept that the turnover of ^13^C-labeled acetate can be quantified during normothermic perfusion of kidneys by detection of the amount of ^13^CO_2_, released in the exhaust gas of the oxygenator^16^. Therefore, we hypothesized that the acetate turnover test during machine perfusion, even at 20 °C, would be an easy and noninvasive method to evaluate kidney grafts and to predict ulterior graft function after transplantation. An objective test that allows to tell transient renal dysfunction from rather permanent renal failure would be of considerable value when aiming to extend the number of available organs for transplantation^17,18^. Here, we propose a noninvasive quantitative method that can measure metabolic turnover of ^13^C-acetate through the citric acid cycle during cell-free, isolated graft perfusion and show that the readout of this diagnostic test exhibits great potential to predict resumption or failing of graft function after actual transplantation.

## Methods

### Investigational device

Quantification of the turnover of our tracer substance ^13^C-acetate during isolated machine perfusion was performed by detection of the metabolic end product ^13^CO_2_ in the gas outlet of the oxygenator using the high precision ^13^CORlab device (ArgosMED, Karlsruhe, Germany).

In contrast to previous devices, which usually only determine the ratio of ^13^CO_2_ to ^12^CO_2_, the ^13^CORLab® measures the absolute concentrations of both isotopes at a sample rate of 1/s using laser-based high precision spectroscopy. This enables a significantly more precise and independent evaluation.

By simultaneous recording the temperature and flow rate of the measured gas, the exact metabolized amount of the ^13^C-labeled molecule can be calculated. This allows the metabolized amount to be calculated in relation to the administered amount of the marker molecule.

The additional continuous measurement of the ^12^CO_2_ concentration increases the robustness of the measurement against drift effects. Using the previously determined base ratio of ^13^CO_2_ to 12CO_2_, a baseline-adjusted ^13^CO_2_ concentration can be calculated. This step compensates for drift effects and ensures that only the actual metabolic process of the marker molecule is represented.

### Porcine kidney grafts

All experiments on isolated kidneys were carried out on organ grafts that were retrieved from dead female pigs, weighing between 30 and 35 kg. Female pigs were used throughout the study to exclude gender variability in the experimental approach and so circumvent putative sex-related confounding disparities in our readouts and reduce the variances within the groups. The number of test animals required could thus be minimized. The donor animal had been euthanized in deep anesthesia by i. v. injection of potassium chloride. The procedure of euthanasia for organ retrieval according to § 4 Abs. 3, TSG (German Legislation on animal protection) has been approved by the responsible authorities (Landesamt für Natur, Umwelt und Klima (LANUK), NRW, Germany). The principles of laboratory animal care (NIH publication no. 85–23, revised 1985) were followed. No Heparin was given at any time.

In order to depict different degrees of graft injury to be evaluated using the method, the organ grafts were removed after different times of warm ischemia (5 min, 30 min, and 60 min) that roughly correspond to good grafts, grafts that are likely to be afflicted with temporary dysfunction and grafts that are usually associated with persistent nonfunction^19,20^. The kidneys were randomly allocated to the respective groups by drawing cards prior to organ retrieval.

After cannulation of the renal artery, the organs were flushed by 100 cm gravity with 100 ml of HTK solution (Köhler Chemie, Bensheim, Germany) on the back-table at 4 °C prior to preservation in HTK overnight for 18 h. Thereafter, the grafts were subjected for an evaluative period of machine perfusion.

### Evaluative machine perfusion

After 18 h of static storage, kidneys from all groups were put on a machine perfusion circuit (Kidney Assist®, XVIVO, Gothenburg, Sweden) and subjected to an end-ischemic machine perfusion of 2 h duration. Perfusion was performed with Belzer MPS solution at 20 °C for 2 h. Thus, no addition of blood components will be required, and oxygenation can safely be performed without the requirements of carbon dioxide as a buffer supplement, which is usually not available for the clinical situation.

Perfusion pressure was regulated by a servo-controlled pump that adjusted the renal flow according to the feedback obtained by a connected pressure transducer in the arterial inflow line. Resulting renal flow values were about 100 ml/min, and oxygenation was provided by an interposed membrane oxygenator fed at a constant flow rate of 0.6 l/min of oxygen. Thus, a largely sufficient tissue aeration at this temperature was ensured according to our own experiences.

### ^13^C-acetate turnover test

During machine perfusion, metabolic measurements on citrate cycle throughput are performed by ^13^C detection in the gas outlet of the oxygenator after bolus injection of 25 mg of ^13^C-acetate. After obtaining steady-state conditions during graft perfusion, the baseline values for ^13^CO_2_ and the ^13^CO_2_/^12^CO_2_ ratio are recorded before application of the tracer. Injection of 25 mg of ^13^C-acetate will result in its metabolization in the renal tissue by the TCA-cycle, yielding ^13^CO_2_ that is released in the venous outflow and subsequently excreted by the oxygenator in the gas outflow line. Thus, it can be detected by a notable rise of the ^13^CO_2_ recording over the baseline level. The amount of substance detected during the first 30 min after injection of the tracer is taken as a readout of the respective metabolic activity of the kidney graft.

### Conventional perfusion parameters

Oxygen consumption was calculated from the pO_2_ differences between arterial and venous sites, measured in a pH-blood gas analyser (ABL 815flex acid-base laboratory, Radiometer, Copenhagen, Denmark) and expressed as ml/100 g/min according to trans-renal flow and kidney mass. Lactate levels in the perfusate were also measured in a pH-blood gas analyser. Alpha glutathione S-transferase (GST) was quantified using a porcine ELISA test according to the manufacturer’s instructions (My Biosource, San Diego, USA).

### Histomorphology

Kidney tissue was collected at the conclusion of the in vitro experiments, cut into small blocks (3 mm thickness) and fixed by immersion in 4% buffered formalin. The blocks were embedded in paraffin, and 2–4 μm tissue slides were prepared using a microtome (HM 325, Microm, Walldorf, Germany). Hematoxylin and eosin (H&E) staining was conducted adherent to in-house standards and used to assess the morphological integrity of the parenchyma.

Assessment was carried out according to Leemans et al.^21^ in 10 randomly chosen, nonoverlapping fields (×400 magnification), using a 3-point scale for tubular dilatation, necrosis, edema, inflammation and fluid in tubules: 0 = no damage; 1 = lesions affecting > 25% of the field; 2 ≥ 50%; 3 ≥ 75%. In each individual kidney, the mean value from the 5 respective parameters was calculated and taken as the individual mean histological injury score.

### Live animal studies/transplantation experiments

Transplant experiments were performed in accordance with the federal law regarding the protection of animals and after approval by the responsible authorities (Landesamt für Natur, Umwelt und Klima (LANUK)), NRW, Germany (AZ 2024-163-GA). The principles of laboratory animal care (NIH publication no. 85–23, revised 1985) were followed. Experiments are reported in accordance to the recommendations in the ARRIVE guidelines (PLoS Bio 8(6), e1000412, 2010). All experiments were carried out on female domestic pigs (German Landrace x Pietrain crossbred), aged between 12 and 14 weeks. The animals were allowed to acclimatize to their surroundings for a minimum of 10 days prior to surgery and had free access to tap water and standard pellet food. They were kept in pairs on a floor with a non-slip epoxy resin coating littered with straw. As enrichment, the animals also receive a hay basket, a permanently mounted wall brush and balls or other chewing equipment in regular rotation per box. The animals were kept at a room temperature of 22 °C. Solid food was withdrawn 20 h before surgery.

A porcine auto-transplantation model was used as established in our laboratory and previously detailed elsewhere^22,23^.

The left kidney was retrieved, preserved overnight and auto-transplanted the next day, subsequent to removal of the native contralateral kidney. Vascular anastomoses were performed end-to-side (renal vein- vena cava) and end-to-end (left renal artery- right renal artery), respectively. At the time of reperfusion, 20 mL of glucose 40% were infused to induce osmotic diuresis. No other diuretics were given. The ureter was cannulated with polyethylene tubing, which is tunneled through the abdominal wall, allowing continuous visual inspection of urine production.

During 7 days follow up, blood samples were taken on a daily basis. Serum levels of creatinine were determined by reflectance photometry on an Element RC3X point of care unit (scil animal care company, Viernheim, Germany).

To reduce the risk of infection, perioperative antibiosis with amoxicillin (Duphamox, 15 mg/kg i.m.) is applied at the onset of the experiment. Pigs were supplied with analgesics (Carprieve, 4 mg/kg i.v.) for the first three days post-transplantation.

Nephrectomy was performed after the donor organ was randomly subjected to 5, 30, or 60 min of warm ischemia by clamping of the renal artery. Randomization was done by drawing cards; four animals were assigned to each protocol. After cold preservation and evaluation by machine perfusion as described above, all grafts were transplanted, and the results of the ^13^C-turnover test on the machine were analyzed in regard to the ulterior renal function after transplantation. Thus, the potential of this parameter to predict kidneys that will or will not regain function after transplantation could be analyzed by receiver operating characteristics curve analysis. Successful recovery of renal function was defined assumed in presence of falling serum creatinine values after at least 4 days. If serum values of creatinine continue to rise the organ was considered a failing graft.

### Statistics and reproducibility

Sample size for the in vitro experiments was calculated for one-way analysis of variance followed by multiple comparison of three groups using BiAS for Windows 10.03 (Epsilon Verlag, Darmstadt, FRG)

According to previous experiences with the isolated kidney model, we assume a mean standard deviation in the experimental procedure of up to 50%.

We expect a minimum range of 1.0 as the relevant difference between the different groups (good, moderate, and poor). These specifications result in a necessary number of trials per group of *n* = 9 for the post-hoc test after multi-group comparison with ANOVA, with a 5% probability of error in the first (alpha) order and a power of 90%.

The sample size calculation in the transplant experiments was based on the ROC curve analysis for the discrimination between kidneys that resume function after transplantation and those that fail to do so by means of the inaugurated ^13^C turnover test during machine perfusion. Thereby, an area under the curve of more than 0.9 was considered as a significant benefit for future use of the parameter.

The rate of type 2 error has been defined as *ß* = 0.2. The level of significance for the type 1 error is set to *p* < 0.05. From these assumptions, it follows that the detection of a discriminative significance compared to the null hypothesis (Area under the ROC curve = 0.5) required a total number of *n* = 12 experiments. (MedCalc® Statistical Software version 20.009 (MedCalc Software Ltd, Ostend, Belgium)); https://www.medcalc.org; 2021).

Values were expressed as means ± SD unless otherwise indicated. Laboratory analyses had been blinded to the experimental groups. Differences between groups were tested by Welch’s one-way ANOVA without assuming equal variances for each group and post-hoc testing using Dunnett’s T3 multiple comparison test. *P* < 0.05 were considered significant. ROC curve data were calculated using Prism 9.01 (GraphPad software Inc., San Diego, CA, USA). No animals were excluded from analyses.

## Results

**Fig. 1:**
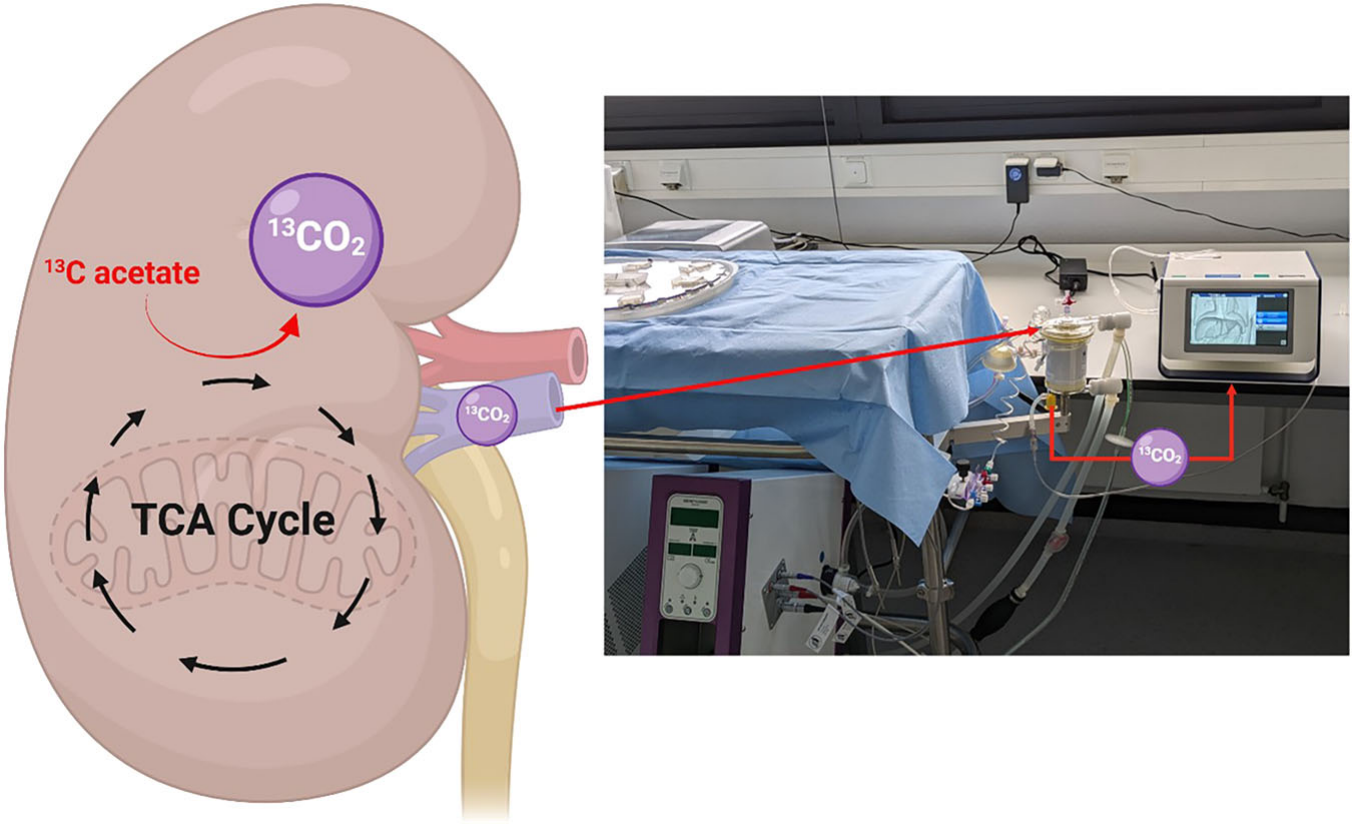
Illustration of the evaluation of acetate throughput in the TCA cycle. After injection of ^13^C-marked acetate into the perfusate, the tracer is metabolized in the TCA cycle to yield ^13^CO_2_, that diffuses into the venous perfusate and passes the oxygenator, where it is evaporated into the gas exhaust line. The gaseous exhaust of the oxygenator is connected to the mobile CORLab device, where the concentration of ^13^CO_2_ is detected at 1 s intervals.

The amount of ^13^C metabolized during renal machine perfusion was measured using the ^13^CORLab from ARGOS MED (Karlsruhe, Germany). The instrument, which enables laser-based, isotope-separated CO_2_ analysis on organs along with simultaneous recording of gas flow and temperature, is easily connected to a standard commercial machine perfusion device, e.g., the XVIVO kidney-assist (cf. Fig. 1).

During machine perfusion of the kidneys at 20 °C with buffered solution, the oxygenator was fed with 100% medical grade oxygen, resulting in adequate oxygenation of the organs as well as a stable pH of the perfusate around 7.203 ± 0.085. The gas flow through the CORLab device averaged to 0.65 ± 0.21 l/min, which lies well within the operation limits of the device (0.015–2.0 l/min).

### Reaction of exhaled ^13^CO_2_ after tracer injection during machine perfusion at 20 °C

**Fig. 2:**
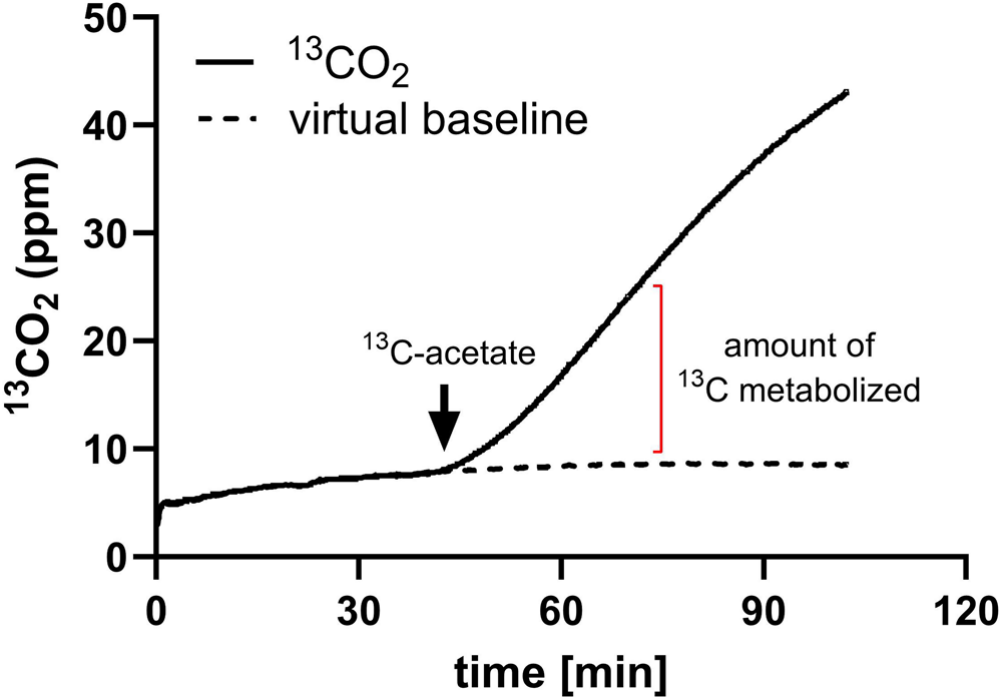
Representation of the detected ^13^CO_2_ at the CORLab device during baseline perfusion conditions and after injection of ^13^C-labeled acetate. A steep rise of the curve is seen shortly after tracer injection that is indicative of the metabolization of acetate by the graft.

Upon initial perfusion of the organs, a baseline signal of ^13^CO_2_ is recorded at the CORLab detector, resulting from the metabolism of the graft and the naturally occurring percentage of ^13^carbon in the environment. When this baseline recording gets stable, the ^13^C-labeled tracer can be injected into the perfusate, which is followed by a steep rise of the ^13^CO_2_ record in the exhaled air, proportional to the metabolic turnover of the ^13^C-moitié of the tracer (cf. Fig. 2).

The turnover of the substrate in the TCA cycle can hence be calculated during the linear rise of the slope from the total gas volume flow and the concentration of the ^13^C-molecules.

At the same time, the concentration of ^12^CO_2_ is continuously monitored and the ratio of ^13^C/^12^C calculated. Using the initially determined ratio at baseline conditions, the baseline-adjusted ^13^CO_2_ concentration is constantly calculated during the measurement process in order to compensate for drift effects. Thus, only the actual metabolic process of the marker molecule is represented. The method provides a precise, drift-corrected, and quantitatively reliable representation of the metabolic process of the marker molecule.

### Redundant measurements

After the first injection of a ^13^C labeled tracer, it takes a certain time until the substance is fully metabolized and a new stable baseline level is reached. This implies a certain lag time before a replicate measurement would be possible.

**Fig. 3:**
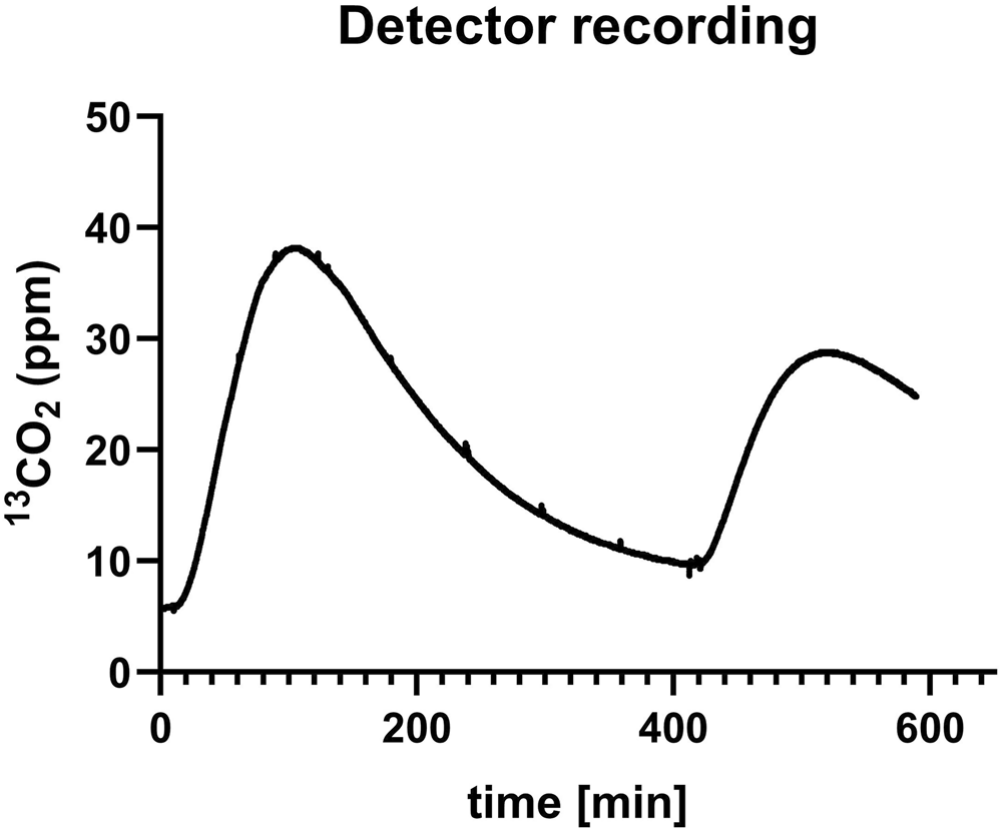
Replicate testing during extended machine perfusion. Recording of consecutive injections of ^13^C labeled acetate as a tracer after 10 and 410 min of machine perfusion.

One kidney graft was kept on the perfusion circuit for more than 10 h in order to evaluate the feasibility of consecutive measurements (cf. Fig. 3). The first measurement was disclosed a renal acetate turnover of 3.6 µmol/30 min.

After the ^13^C-recording has turned back to approximately baseline values after a total time of 470 min of perfusion, a second tracer injection was performed. The second measurement resulted in a metabolic turnover of 3.5 µmol of ^13^C per 30 min.

From these data, it follows that approximately 5 h should ideally lie between two consecutive measurements under normal conditions.

The interval of approximately 5 h should generally be considered adequate for re-evaluation of marginal kidney grafts after interposed therapeutic or regenerative measures, aiming to restore graft transplantability.

If, however, a measurement has to be repeated before the ^13^C recording has reached a new baseline level, the respective baseline level must be corrected for the amount of ^13^C remaining from the previous tracer injection.

**Fig. 4:**
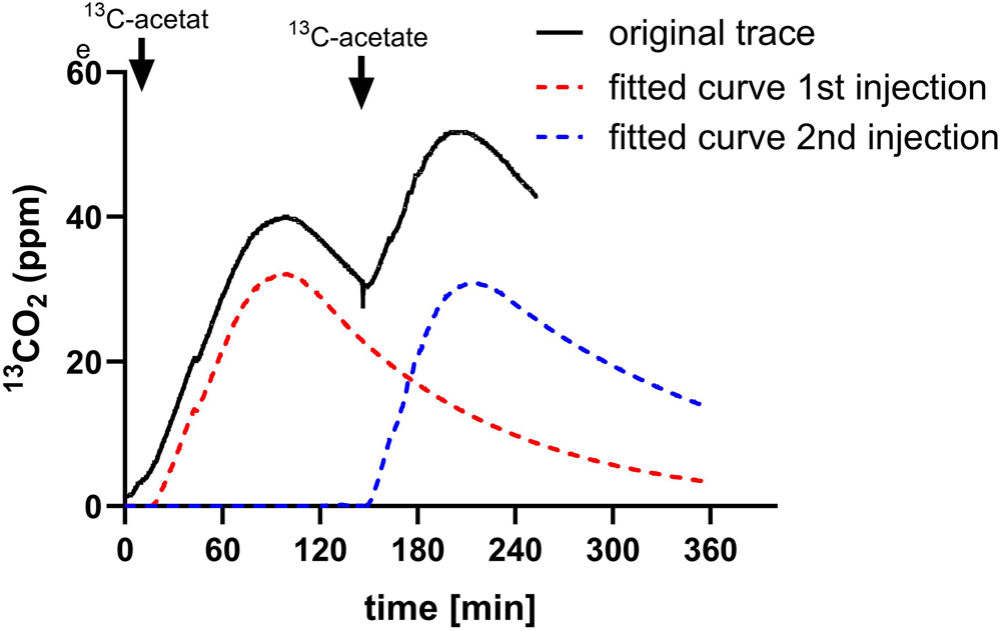
Schematic representation of curve fitting for evaluation of a second tracer injection prior to complete metabolization of the first dose. Original trace depicted as a solid black line. Red line shows the fitted curve representing only the first injection by approximation through a e-function: ^13^CO_2_ = *c*(*t*_o_) × e^−*k*^^(^^*t − t*^^o^^)^. The approximated isolated curve for the second tracer injection is obtained by subtracting the red line from the original recording, as represented by the blue dotted line. Calculated results for the ^13^C metabolization were 7.0 µmol/30 min for the first measurement and 6.8 µmol/30 min for the second measurement.

This can be done by extrapolation of the declining ^13^C curve by curve fitting and continuous subtraction of this virtual baseline from the actual reading after a second injection of tracer substance (see Fig. 4).

Even though a second reading is thus possible already 1–2 h after the first read-out, this procedure is affected by a somewhat reduced accuracy, due to the requirements to approximate a declining baseline amount of ^13^C.

### Renal function assessment

In order to investigate how accurately the metabolic turnover of ^13^C acetate at 20 °C reflects actual differences in renal graft integrity, porcine kidneys were retrieved after varying times (5, 30, or 60 min) of warm ischemia in the donor animal, perfused with HTK preservation solution and cold stored overnight for 18 h.

The other day, all grafts were subjected to an evaluative machine perfusion at 20 °C for 2 h, while the gas outlet from the oxygenator of the perfusion system was connected to the CORLab detector. The amount of ^13^CO_2_ detected by the system during 30 min after injection of the labeled acetate tracer was measured for each kidney, and the mean values for each of the three groups were compared by ANOVA and appropriate post hoc testing.

**Fig. 5:**
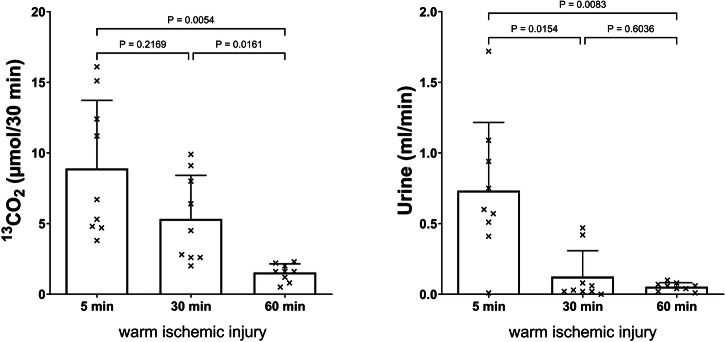
Markers of kidney function during machine perfusion. Differences between functional markers of renal integrity obtained during machine perfusion of kidney grafts subjected to 5, 30, or 60 min of warm ischemic injury prior to cold storage, respectively. Data are given a mean ± SD and individual values of *n* = 9 per group; Welch’s one-way ANOVA and post-hoc testing using Dunnett’s T3 multiple comparison test.

It was seen that the group with 60 min of warm ischemia significantly differed from both other groups, while the difference in metabolization of ^13^C-acetate did not reach significance between the groups of 5 of 30 min of warm ischemic injury (Fig. 5).

By contrast, renal production of urine differed significantly after 5 or 30 min of warm ischemia, but did not disclose any discriminative potential between 30 and 60 min of warm ischemia, as the kidneys turned out to be oligo- to anuric in both groups.

Of note, kidneys after 30 min of warm ischemia often regain function in porcine renal transplant experiments, although frequently delayed by a certain time of anuria^19,24^, whereas grafts that had suffered 60 min of warm ischemia will, in the great majority, end up in long-term dysfunction and ultimate graft loss^25,26^.

In consequence, the distinction between the latter two groups would be of paramount importance when it comes to decide, whether or not a questionable kidney graft should be transplanted or rejected.

Other parameters, like lactic acid accumulation or oxygen consumption during machine perfusion, did not help to solve this challenge (lactate: 0.58 ± 0.26 vs. 0.59 ± 0.10 vs. 0.69 ± 0.12 (*p* = 0.2346 vs. 30 min) µmol/l; oxygen consumption: 1.69 ± 0.37 vs. 0.93 ± 0.50 vs. 1.21 ± 0.40 (*p* = 0.5520 vs. 30 min) ml/min/100 g; 5 min vs. 30 min vs. 60 min warm ischemia).

### Histomorphology

Histological examination revealed rising injury score levels along with the extension of warm ischemic injury, mainly due to increased prominence of tubular dilatation and cellular oedema in the more severely damaged groups. Mean score values after 5 min of warm ischemia (0.26 ± 0.17) differed significantly from those after 30 min (0.53 ± 0.14; *p* = 0.008) and after 60 min of warm ischemia (0.71 ± 0.18; *p* = 0.0002). The difference between the two latter groups did not reach statistical significance.

### Porcine kidney transplantation

As final validation of the ^13^C-turnover measurement to be a useful indicator in determining the acceptability of a graft for transplantation, we set up a series of experiments with actual renal transplantation in vivo.

Twelve kidney grafts were subjected to varying degrees of warm ischemic challenge upon retrieval (5, 30, or 60 min), preserved overnight by cold storage and finally evaluated by 2 h of machine perfusion at 20 °C prior to actual re-transplantation in the nephrectomized donor animal.

Renal function after transplantation was defined by decreasing daily serum creatinine values after at least 4 days post-implantation, whereas non-function was stated in the presence of rising serum creatinine values over at least 5 days. Moreover, if serum creatinine values exceeded 13 mg/dl, the animal had to be euthanized in accordance with ethical obligations given by the animal ethics committee. If this occurred prior to postoperative day 4, the kidney graft was also classified as not functioning.

The individual results of the ^13^C-metabolization test on the machine as differentiated between

**Fig. 6:**
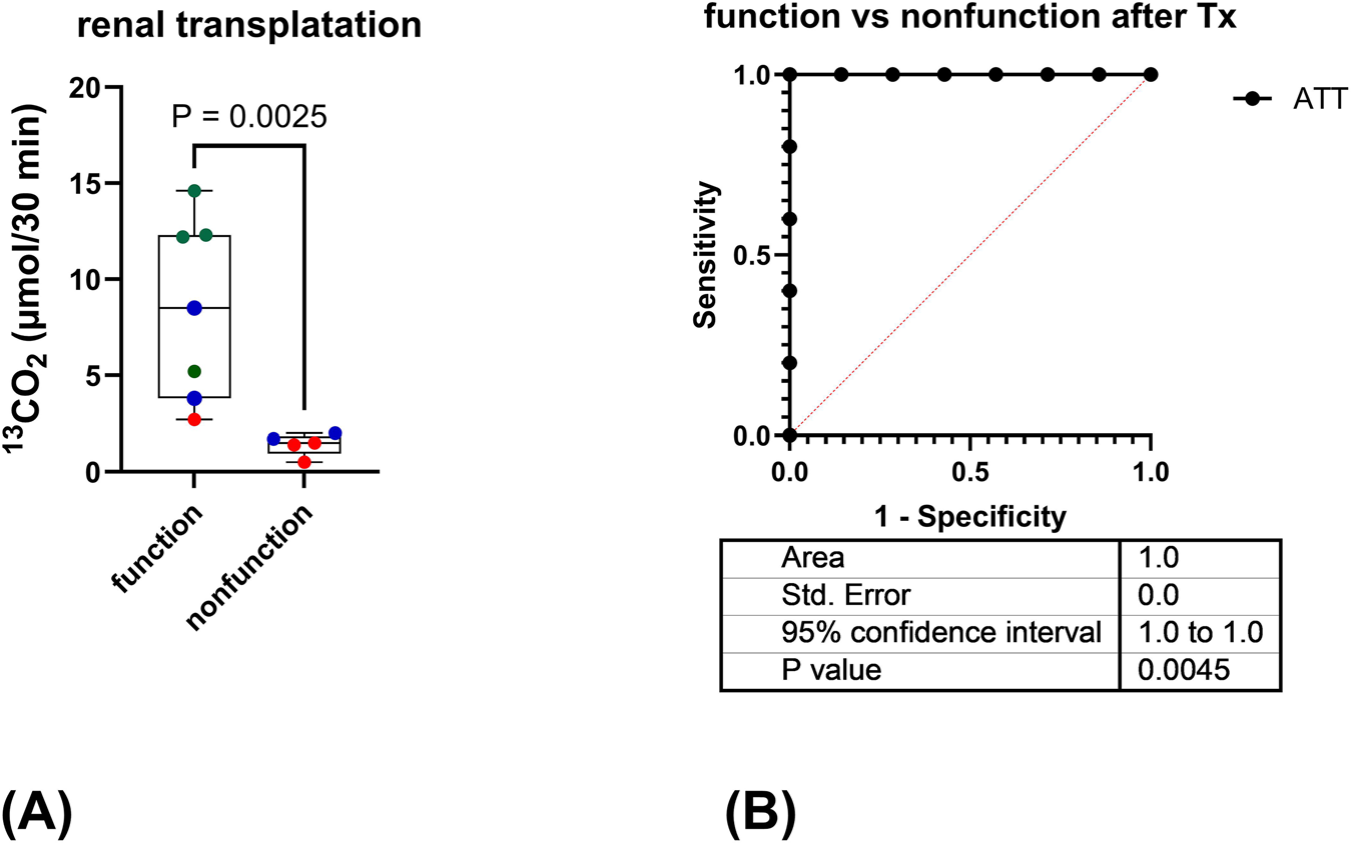
^13^C turnover and results after renal transplantation. **A** Differences between the ^13^C turnover of kidney grafts during machine perfusion, depending on whether the graft resumed function after subsequent transplantation. Data are given a median ± min/max and individual values of *n* = 7 (functioning) and *n* = 5 (non-functioning) grafts, respectively. *P* = 0.0025 by comparison with the Mann–Whitney *U*-test. **B** Receiver-operating characteristics curves illustrating the accuracy for ^13^C-acetate metabolization observed during machine perfusion to allow for discrimination between renal grafts that did or did not resume function after subsequent transplantation.

Kidney grafts that resumed function after transplantation and those that did not are depicted in Fig. 6A. Of note, permanently non-functioning kidneys had significantly lower turnover rates of the tracer than the grafts that were successfully transplanted and no overlap in the individual values was seen between the two groups.

Accordingly, the receiver operating characteristics curves of ^13^C-acetate metabolization for discrimination between functioning and non-functioning grafts actually disclose an area under the curve (AUC) of 1.0, indicating a significant (*p* = 0.0045) discriminative potential to separate acceptable from non-acceptable kidneys prior to actual transplantation (cf. Fig. 6B).

Several conventional parameters that are frequently used for graft evaluation during machine perfusion have been evaluated for comparison (cf. Table 1). Best results were obtained for perfusate levels of alpha-glutathion transferase with an area under the ROC curve of 0.83, but in contrast to the results of the ^13^C test, all of the tested parameters did fail to disclose a significant discrimination between functioning and failing kidney grafts.

**Table 1:** Area under the receiver operating characteristics (ROC) curve, 95% confidence interval (CI) and level of significance to discriminate between functioning and failing kidney grafts by some conventional parameters obtained upon subnormothermic machine perfusion

	Area	95% CI	*P* =
Renal flow (ml/min/g)	0.77	0.48–1.0	0.123
Lactic acid (mmol/L)	0.51	0.17–0.86	0.935
Oxygen consumption (ml/100 g/min)	0.70	0.39–1.0	0.256
Glutathione-S-transferase (ng/ml)	0.83	0.58–1.0	0.062
Urine production (ml/min)	0.77	0.48–1.0	0.123

## Discussion

We have described a method for noninvasive evaluation of metabolic turnover at the tricarboxylic acid cycle (TCA) in kidney grafts as a parameter that represents overall functional integrity of the organ.

The test can easily be performed during extracorporeal machine perfusion of the graft prior to transplantation, and the results have been shown to be highly predictive of ulterior graft recovery after engraftment in vivo.

In the face of a still elevated rate of graft failure in extended criteria donor organs, the proper evaluation of kidney graft quality represents a paramount challenge in transplantation medicine^27^, that continues to gain importance, as donor organ shortage urges to increase of the percentage of grafts used for transplantation.

While confirmation of renal integrity prior to engraftment is mandatory to safeguard patient health, a non-substantiated rejection of functionally acceptable grafts must be avoided in order to optimize the usage of the scarce donor pool.

In this context, the diagnostic focus lies in sorting out of permanently failing grafts from the pool of acceptable ones that will regain function.

During the last decade, a variety of perfusate biomarkers have been investigated as estimate of renal integrity during hypothermic or normothermic machine perfusion. Although some of them (e.g., alpha GST, NGAL, or H-FABP) did present an independent association with post-transplant renal outcome (e.g., glomerular filtration after 6 months), their predictive accuracy was only low to moderate, and none of them could reliably predict ulterior graft function^28,29^.

The group of Hosgood and coworkers pioneered in developing a scoring system, intended to evaluate kidney grafts during normothermic ex vivo machine perfusion^30^. They looked at macroscopic appearance, renal flow and urine production to eventually categorize the grafts for transplantability.

But then, although higher urine production during machine perfusion is associated with adequate kidney function, many initially anuric or oliguric kidneys might regain function later on and qualify for transplantation. Moreover, others did not find any robust correlation between urine production during machine perfusion and graft function after transplantation^31^.

Recently, the analysis of renal vascular perfusion by magnetic resonance imaging (MRI) has come into focus to assess kidney grafts prior to transplantation. During normothermic perfusion in a MRI-compatible machine, it became evident that largely injured porcine kidneys exhibited significantly altered corticomedullary blood flow distribution with respect to minimally injured grafts or the in vivo situation^32^. However, no data were yet available from mildly injured, hence potentially acceptable renal grafts.

In our study, we tried to evaluate the metabolic performance of the kidneys by measurement of acetate turnover during machine perfusion. Acetate is a metabolite that is easily taken up from the circulation by the kidney and directly metabolized through the TCA cycle^33^.

The radioactive ^11^C-acetate is a well-known tracer for the study of myocardial oxidative metabolism using positron emission tomography (PET)^34^.

Likewise, the non-radioactive ^13^C-acetate has successfully been used to investigate intrarenal energetic turnover in vivo using MRI^35^.

Our approach to simply measure ^13^C-carbon in the gas outlet of the oxygenator benefits from the fact that, due to the isolated perfusion approach, any metabolized carbon can be referred to the kidney, thus precluding the need for topical analyses and the availability of an MRI device. The transportability of the CORLab device makes this technology easily available at any place where machine perfusion of donor grafts might be performed.

A primary validation of the acetate turnover test to be actually associated not only with different degrees of ischemic tissue injury but actually linked to post-transplant resumption of graft function can be derived from the missing overlap in the results from functioning and non-functioning grafts, leading to the significant area under the ROC curve.

Although the discriminative potential of the described technique to adequately reflect different degrees of renal injury is also operational at normothermia^16^, we decided to perfuse the organs at mid-thermia in order to circumvent the need for red blood cells as additional oxygen carriers, which is often claimed necessary for organ perfusion at normothermic temperatures^36,37^.

Unlike the situation in the liver, where free hemoglobin is converted into bilirubin and excreted via the biliary system, invariable hemolysis during erythrocyte-based perfusion of the kidney will lead to hemoglobin-mediated tissue injury^38^.

Additional drawbacks might be seen in the increased risk of microbial contamination and the acute challenge of warm ischemic injury if the device fails inadvertently^39^. Therefore, machine perfusion of individual donor kidneys is often performed at lower temperatures^10^, which also significantly simplifies the technical requirements as well as the demands on the perfusion medium^11,12^.

However, assessment of graft viability below normothermic temperatures had not yet been well defined^9,14,15^. The described technique to evaluate acetate turnover in the TCA cycle at 20 °C closes this diagnostic gap. Although the absolute metabolic flux through the TCA cycle is sensibly lower than at normothermia (cf.^16^), the relative discriminative power of our test was also obvious at 20 °C and outperformed the conventional parameters investigated in our study.

As a matter of fact, a clinically relevant cut-off value for the ^13^C turnover test cannot be derived from our relatively small experimental cohort. Adequate threshold values for the ^13^C turnover test, as well as final prove for its general value, have to be established upon a clinical pilot study also including issues of renal dysfunction other than ischemic injury.

Nevertheless, the test combines ease of use and real-time availability, and might become a valuable tool helping the transplant surgeon in deciding whether to use or to discard an individual donor graft.

## Supplementary information

Description of Additional Supplementary Files

Supplementary Data 1

## Data Availability

The source data for Figs. 5 and 6 can be found in Supplementary Data 1. All other data are available from the authors upon reasonable request. (c/o C. v. Horn: charlotte.von-Horn@uk-essen.de).
