# Supplementary material for: A metabolic test to distinguish temporary and persistent renal graft dysfunction prior to transplantation
Source: Commun Med (Lond). 2026 Jul 9;6:388. doi: 10.1038/s43856-026-01736-x (PMC13350869; doi:10.1038/s43856-026-01736-x)
Supplement: Supplementary file 1 — Description of Additional Supplementary Files [file 43856_2026_1736_MOESM1_ESM.docx]

Description of Additional Supplementary Files

File name: Supplementary Data 1

Description: The source data for the figures 5 and 6
